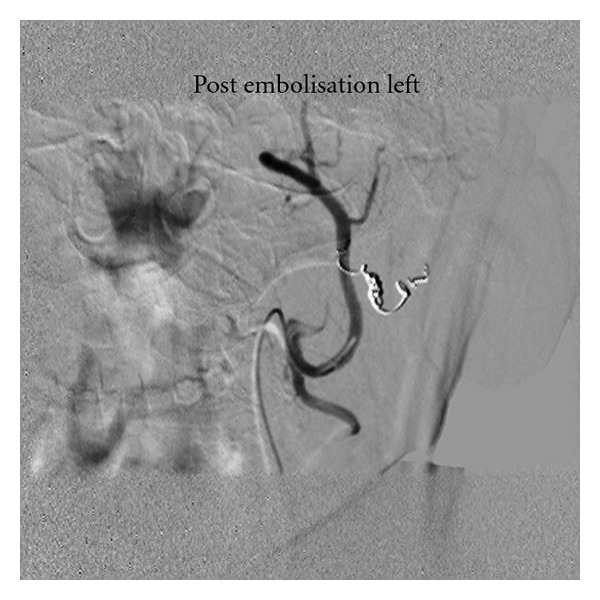# Endovascular Treatment of a Giant Aneurysm of the Maxillary Artery

**DOI:** 10.1155/2011/818241

**Published:** 2012-01-24

**Authors:** J. A. Stephenson, S. Panteleimonitis, E. Choke, M. Dennis, M. Glasby

**Affiliations:** Department of Vascular Surgery and Department of Radiology, University Hospitals of Leicester, Leicester Royal Infirmary, Infirmary Square, Leicester LE1 5WW, UK

## Abstract

Aneurysms of the maxillary artery are rare and the majority of the literature refers to false aneurysms. We report the first case of what we believe to be a spontaneous true maxillary artery aneurysm and its endovascular management.

## 1. Introduction

Aneurysmal enlargement of the maxillary artery is extremely rare [1, 2]. The current literature on these aneurysms generally relates to false aneurysms occurring secondary to direct penetrating or blunt trauma, or from an iatrogenic injury [3–6]. There are no reported cases of true aneurysmal enlargement involving all three layers of the artery wall. We report a case of a true maxillary artery aneurysm and discuss its management and treatment.

## 2. Case Report

A 75-year-old gentleman of Indian origin presented to a regional Ear, Nose, and Throat Department as a tertiary referral. The history of a painful lump over the left side of his upper neck was for approximately one year. The patient reported that the lump fluctuated in size and was initially painful but this improved with time. The accuracy of history was limited by the fact that the gentleman had a thick beard and may not have noticed the enlargement during shaving or from the external appearance. The pain was intermittent but worse after talking for long periods. He did not have any difficulty eating, shortness of breath, or alteration of hearing. There was no history of preceding trauma. Otherwise the patient was in good health, with no significant comorbidities.

On examination the lump was located in the level 2 area of the neck extending into the preauricular area. The mass was firm and pulsatile. There was no facial nerve weakness or paralysis.

**Figure 1:**
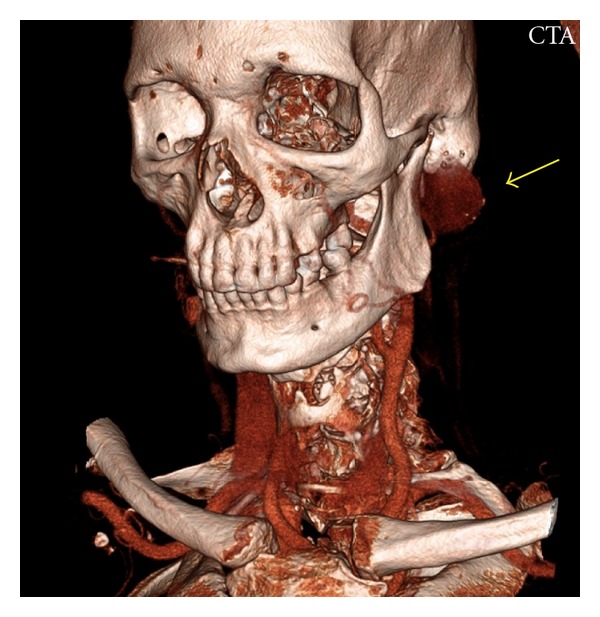


**Figure 2:**
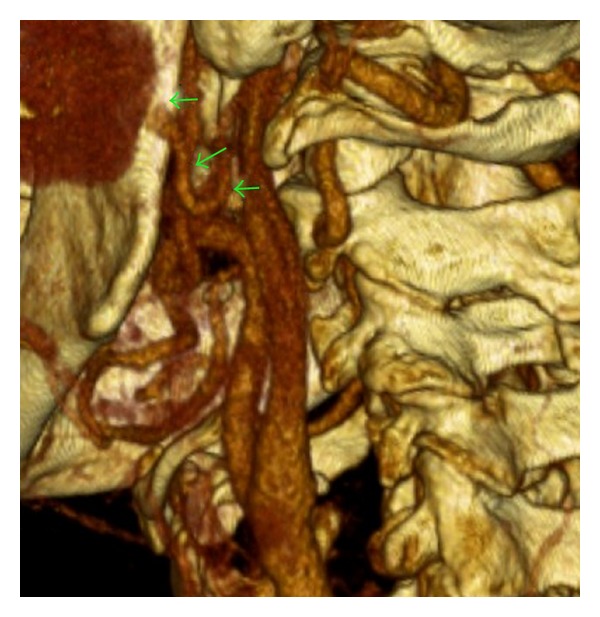


All routine blood investigations, which included full blood count, renal function, clotting, C-reactive protein, liver function, fasting glucose, and lipids, were within normal limits. An ultrasound scan (US) of his left parotid region demonstrated a large aneurysm measuring 45 mm in maximum diameter, closely related to the left parotid gland. A subsequent computed tomographic (CT) angiogram was performed (Figure (1)). This showed a thick walled aneurysm superficial to the mandible arising from the maxillary branch of the external carotid artery (Figure (2)). Doppler US of his aorta and popliteal vessels excluded any further concurrent aneurysms.

After discussions in the vascular surgical multidisciplinary meeting and with the patient it was decided to attempt endovascular embolisation.

**Figure 3:**
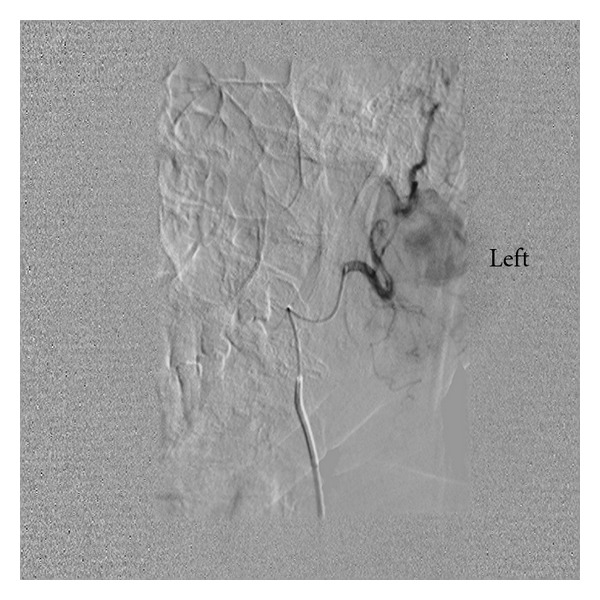


The external carotid artery was selectively catheterised with a 5 French vertebral catheter. Angiography during the procedure showed a very tortuous maxillary artery and the giant aneurysm arising from its midportion. The outflow branches appear to be arising from the sac itself and therefore we concluded that this was a true aneurysm. A microcatheter (Progreat-Terumo, Japan) was used to superselectively embolise the outflow vessel and the aneurysm inflow with (Vortex Coils-Cook Inc, USA) coils (Figure (3)). Immediate angiography showed a successful procedure with no demonstrable flow in the aneurysm (Figure (4)). Subsequently Doppler US of the aneurysm was performed which confirmed that there was thrombus and no flow in the aneurysm sac. There were no postprocedure complications and the patient was safely discharged home the following morning. At 6-month follow-up the mass has disappeared on clinical examination. Doppler ultrasound shows a tiny (<5 mm) area of residual flow at the aneurysm neck. No further treatment is planned.

## 3. Discussion

Aneurysms in the extra cranial portion of the external carotid are extremely rare. There are only 36 published case reports or series of aneurysms of the maxillary artery. The first description was by Kutzleb [1] in 1952. In the majority of these reports the aneurysmal enlargement of the maxillary artery is false or pseudoaneurysmal in nature and secondary to either direct penetrating or blunt trauma or an iatrogenic injury. There are no reported cases of true aneurysmal enlargement. 

El-Sabrout and Cooley [2] published a case series in 2000 in which 7394 aneurysms were examined between 1960 and 1995. Of those only 67 (0.9%) involved the external carotid artery. Of those 67 only 23 (34%) were atheromatous in nature. None of those reported were located in the maxillary branch of the external carotid artery. 

More recently, in 2008 Masoomi et al. [3] reported the case of a traumatic false aneurysm of the internal maxillary artery in 5-year-old girl treated by operative surgery due to lack of endovascular facilities. However in the majority of recent case reports in the medical literature of maxillary artery aneurysms, endovascular embolisation has been the treatment modality of choice. In 2010, Chepla et al. [4] described a case of maxillary artery false aneurysm after Le Fort I osteotomy which was treated using transcatheter arterial microcoil embolisation. Similarly in 2007, Silva et al. [5] describe a maxillary artery false aneurysm following sagittal split ramus osteotomy. In this case the anatomy was confirmed with CT angiography and treated with percutaneous coil embolisation and thrombin injection. The same year, a similar case was reported following a stab wound, which was treated with coil embolisation with no associated complications [6]. 

Ultrasonography is the most common noninvasive test available to diagnose masses in the neck. However, multislice CT has the advantage of giving detailed anatomical information and for screening other body regions for coexisting aneurysms. It was previously suggested that diagnosis of maxillary artery aneurysms depended solely on catheter angiography, as precise CT detection was difficult [7]. However, with more advanced CT angiographic technology, CT was shown to be adequate for diagnosis and treatment planning in this case, adequately defining the location, origin, and extent of the lesion. 

Treatment options for these aneurysms include endovascular embolisation or surgical excision. Percutaneous embolisation is safe, quick, and an effective technique for such lesions and embolisation in the head and neck area is a frequently utilised therapy for epistaxis [8] and post-traumatic haemorrhage [9, 10]. Although no complications arose from the endovascular procedure in this instance, the expected possible complications of maxillary artery embolisation would be the same as those of embolisation for epistaxis. These can be categorized as minor transient (reported rates of 25%–59%), major transient (0%-1%), or persistent (<2%) [11–16]. Minor transient adverse effects include headache, facial pain, jaw pain, facial oedema, parasthesia, palate ulceration, and trismus. Major transient complications include skin sloughing, temporary hemiparesis, temporary monocular visual field loss, and mucosal necrosis. Persistent complications include monocular blindness, peripheral facial nerve paralysis, and cerebral infarction. Complications related to vascular access must also be considered [11–16]. However, embolisation offers the advantage of being able to reach vessels that would be difficult to approach surgically [4] and generally has lower reported complication rates. In our centre, surgical exploration and excision is reserved as a last result for cases where embolisation has failed or in cases of aneurysms with signs of local infection [5]. 

## Figures and Tables

**Figure 4:**